# Healthcare professionals’ experiences of providing individualized nutritional care for Older People in hospital and home care: a qualitative study

**DOI:** 10.1186/s12877-019-1339-0

**Published:** 2019-11-20

**Authors:** Christine Hillestad Hestevik, Marianne Molin, Jonas Debesay, Astrid Bergland, Asta Bye

**Affiliations:** 1 Department of Physiotherapy, Faculty of Health Sciences, OsloMet - Oslo Metropolitan University, Oslo, Norway; 2 Department of Nursing and Health Promotion, Faculty of Health Sciences, OsloMet - Oslo Metropolitan University, Oslo, Norway; 3 Bjorknes University College, Lovisenberggata 13, 0456 Oslo, Norway; 4 Regional Advisory Unit in Palliative Care, Department of Oncology, Oslo University Hospital, Oslo, Norway

**Keywords:** Nutritional care, Older persons, Interviews, Healthcare professionals, Hospital, Home care, Organization of care

## Abstract

**Background:**

Recent studies indicate inadequate nutritional care practices in healthcare institutions and identify several barriers to perform individualized nutritional care to older persons. Organisation of care can become rigid and standardised, thus failing to be respectful of and responsive to each person’s needs and preferences. There is limited research exploring health professionals’ views on how structure of care allows them to individualize nutritional care to older persons. In this study we aim to explore how healthcare professionals’ experience providing individualised nutritional care within the organisational frames of acute geriatric hospital care and home care.

**Methods:**

Semi-structured interviews with 23 healthcare professionals from hospital acute geriatric care and home care. Interviews were analyzed using thematic analysis.

**Results:**

Two main themes and six sub-themes emerged from the material. Theme 1: ‘Meeting patients with complex nutritional problems’ with the sub-themes: ‘It is much more complex than just not eating’ and ‘seeing nutrition as a part of the whole’. Theme 2: ‘The structure of the nutritional care’, with the sub-themes: ‘Nutritional routines: Much ado, but for what?’, ‘lack of time to individualize nutritional care’, ‘lack of interdisciplinary collaboration in nutritional care’ and ‘meeting challenging situations with limited resources in home care’.

**Conclusions:**

The healthcare professionals described having a high focus on and priority of nutritional care when caring for older persons. They did however find it challenging to practice individualized nutritional care due to the complexity of the patients’ nutritional problems and constraints in the way nutritional care was organised. By describing the challenges the healthcare professionals face when trying to individualize the nutritional care, this study may provide important knowledge to health professionals and policy makers on how to decrease the gap between older patients’ preferences for care and nutritional care practice.

## Background

Over the past 10 decades, studies have reported a high prevalence of malnutrition and inadequate nutritional care practices in healthcare institutions; in spite of these findings, the problem has persisted [1]. In 1999, the Council of Europe established a network of national experts within nutrition from eight member states in order to improve practices. The aim was to issue clinical guidelines (i.e. a framework for decisions supporting best practices to manage the risk of malnutrition in order to improve treatment outcomes) [1]. As a result, a number of European countries, including Norway, have published national guidelines on the treatment and prevention of malnutrition to ensure high-quality nutritional care throughout the continuum of care [2–8].

Several studies exploring nutritional care practices report a significant gap between recommended nutritional care and actual practices in many healthcare facilities [9–14]. Researchers have identified problems such as lack of knowledge and priority among healthcare professionals [12, 14–16], institutional constraints, uncertainty about roles and responsibilities, lack of allocated time and lack of communication between professionals and services as possible causes [9, 10, 12, 13, 16]. In addition, a recent study found that healthcare professionals experienced a conflict between respecting a patient’s autonomy and practicing standardised nutritional care [14]. The nurses expressed concern that the individual patient was forgotten in the standardisation of nutritional care, as the treatment was not based on the patient’s particular needs and preferences. Healthcare services are complex and, the organisation of work may become rigid and compartmentalised, thus failing to do justice to the richness of people’s lives as well as the complex nature of specific health problems [17]. Healthcare professionals’ personal values and beliefs about what constitutes good nutritional care may conflict with the care they are able to deliver within these institutional constraints, resulting in a sense of inadequacy about their work. In addition, they are confronted with pressure from clients/patients with infinite needs [18].

In spite of the literature indicating little change in practice with regard to nutritional care, there is limited research exploring healthcare professionals’ views on how the organisation and standardisation of care allows them to individualise the nutritional care they provide to older patients. Thus, in this study we aim to explore how healthcare professionals’ experience providing individualised nutritional care within the organisational frames of acute geriatric hospital care and home care.

## Methods

### Design

We used a qualitative design with an interpretive descriptive approach [19]. We conducted face-to-face individual interviews to gather descriptions of the diversity and nuances in the healthcare professionals’ views on the nutritional care practice, to increase our understanding of this complex phenomenon. Interviews were transcribed, and analyzed using thematic analysis [20].

### Sampling

Participation required that the healthcare professionals were involved in providing nutritional care to older patients and that they held a minimum 50% position at a geriatric hospital ward or in home care services.

We sent information about the project and a request for participation to the managers of all the 15-home care districts and to the nursing managers of the three largest geriatric hospital wards in a large city in Norway. Five units within home care and two hospital wards responded and agreed to participate. The first author then arranged a meeting with these units and wards, where she introduced herself as a PhD candidate with an education in nutrition, informed the participants about the study and told them that participation was voluntary. She presented the participants with verbal and written information about the purpose of the study and what participation would entail.

In total, we invited 24 healthcare professionals to participate. Two declined, two withdrew because of illness and two withdrew because of problems finding a suitable time to conduct the interview, resulting in 18 included participants. An additional five participants, two from hospitals and three from home care, were recruited by their nursing managers instead of by the authors. This was due to difficulties finding time for a group meeting where the first author could inform them about the study and ask them if they would like to participate. We aimed to recruit 25–30 participants or continue recruitment until we reached data saturation [21].

### Interview guide

We used a semi-structured interview guide to direct the interviews toward the participants’ experiences with nutritional care by asking open-ended questions; we asked probing questions to clarify aspects that came up in the interviews. Examples of the interview questions are shown in Table 1. The interview guide is included in Additional file 1.

**Table 1:** Examples of questions from the interview guide

Can you please tell me how you perceive older patients’ needs for nutritional care?	
Can you describe how you practice nutritional care?	
How do you perceive the quality of the care you provide?	
How do you experience that the patients’ needs and preferences are met in the nutritional care you provide?	
Can you describe factors that facilitate or prevent you from providing patients with nutritional care according to their needs?	

### Setting

The acute geriatric hospital wards included in this study provide specialised aged care using multidisciplinary team approaches to improve patient outcomes. Their specialty is examining and treating disorders that are common among older patients, including acute and chronic diseases, preventive work, rehabilitation and end-of-life care, including palliative treatment.

Home care services are administered to people who require home healthcare services for either a short or long period because of illness, impaired health, old age or other factors. The care includes practical help with hygiene, cooking and cleaning and home nursing (medication, wound treatment, nutritional care and guidance).

Municipalities administer home care services in Norway. When we carried out our study, the municipal services in Norway were mainly based on the so-called ‘purchaser–provider model’, where the administration and provision of services are split into two separate units. Formal agreements (contracts) and standardised help guide the relationship between home care service staff and care receivers. An administration unit that provides information and specifies contracts assesses a potential care receiver’s needs for care (time and type of care). The administration then orders the services from a provider unit that delivers services as specified in the needs assessment [22, 23]. In the city where we conducted our study, town district units organised home care services.

### Data analysis

We audiotaped and transcribed all the interviews, reviewed the transcriptions for accuracy and anonymised them. We used the software package NVivo for data management, coding and data analysis. We used a thematic coding technique based on Braun and Clarke’s [20] work; it is a method for reporting patterns within qualitative data that includes six phases: [1] familiarising yourself with the data, [2] generating initial codes, [3] searching for patterns or themes across data, [4] reviewing themes, [5] defining and naming themes [6]; writing the report. Examples of the coding strategy is presented in Table 2.

**Table 2:** Examples of the coding strategy

Unit meanings	Initial codes	Categories	Sub-themes	Themes
‘I have tried to talk to undernourished patients about the importance of eating, and sometimes I understand that it is much more complex than just not eating – it is because they don’t want to live anymore and want to let go’ (GHC 8).	Meeting undernourished patients in difficult situations	The patients situations	It is much more complex than just not eating	Meeting patients with complex nutritional problems
‘I see that when the patients get medication for nausea, we get much further with the nutritional care treatment’ (HC 6).	Treating underlying symptoms helps	Other factors that affect nutrition	Seeing nutrition as a part of the whole	Meeting patients with complex nutritional problems
‘We don’t always have the time to ask what the patient wants. We just serve something and are too busy to check whether the food gets eaten’ (GHC 9).	Lack of time to involve patients	Time constraints	Lack of time to individualize care	The structure of the nutritional care
‘There is no cooperation. I don’t even know who the dietician employed by the municipality is’ (HC 1).	No available dietitian	Lack of interdisciplinary support	Lack of interdisciplinary collaboration in nutritional care	The structure of the nutritional care

To maximise trustworthiness and limit threats to validity, we employed the criterion for ‘trustworthiness’ that Lincoln and Guba [24] outlined. We enacted the criterion for credibility through open-ended questioning, spending a prolonged amount of time with the material and giving a detailed description of the methods. To meet the criterion for transferability, we present detailed and in-depth descriptive data and quote the participants in the text. To meet the criterion for dependability, each transcription was independently read, checked and coded by two of the authors; we reached final interpretations through agreement among all five authors. To meet the criterion of confirmability, we present rich quotes from the participants that depict each theme. We also follow the consolidated criteria for reporting qualitative studies (COREQ) [25].

## Results

### Participants

In total, 23 healthcare professionals (22 women and 1 man) participated in this study: 12 healthcare professionals from 2 geriatric hospital wards and 11 healthcare professionals from 5 district units within home care. The average age of the hospital staff was 28 years, and the average time of employment was 3 years. The average age of the home care staff was 41 years, and the average time of employment was 7 years (Table 3). The first author carried out individual interviews between February and April 2018. The interviews took place at the participants’ workplaces and lasted between 33 and 110 min. After 23 interviews, no new insights relevant to the study topic seemed to be revealed, and data was considered to have reached saturation.

**Table 3:** Characteristics of the participants

Acute Geriatric Hospital Care (GHC)				Home care (HC)			
*Informant*	*Profession*	*Position (%)*	*Time on the ward (years)*	*Informant*	*Profession*	*Position (%)*	*Time in the unit (years)*
1	Nurse	58	0.5	1	Nurse	100	1
2	Nurse	80	1.5	2	Nurse	75	5
3	Nurse	80	10	3	Activity therapist	100	17
4	Nurse	80	3	4	Nurse	100	5
5	Nurse	80	0.8	5	Nutrition nurse	100	2
6	Nurse	100	6	6	Nurse	100	9
7	Nurse	50	1.5	7	Nurse	100	6
8	Nurse	80	1.5	8	Nurse	100	7
9	Nurse	100	1.3	9	Nurse	100	25
10	Nurse	80	2.5	10	Nurse	85	2
11	Nurse	100	2	11	Nurse	100	2
12	Nurse	80	3				

## Findings

Our final analysis resulted in two main themes and six sub-themes, as described in Table 4.

**Table 4:** The main themes and sub-themes emerging from the interviews

Meeting patients with complex nutritional problems	It is much more complex than just not eating Seeing nutrition as a part of the whole
The structure of the nutritional care	Nutritional routines: Much ado, but for what? Lack of time to individualize nutritional care Lack of interdisciplinary collaboration in nutritional care Meeting challenging situations with limited resources in home care

### Meeting patients with complex nutritional problems

#### It is much more complex than just not eating

The healthcare professionals in hospital and home care described providing nutritional care to older patients as challenging because nutritional problems are often multifactorial and complex. They experienced that physical, psychological and social issues like dental problems, dysphagia, addiction, dementia, depression and loneliness affected the patients’ appetites and ability to eat. These problems also affected the patients’ willingness to eat:

‘From the conversations I have had with the patients, it seems like the lack of food intake is not solely due to illness, but it is also due to more psychosocial issues like the loss of a loved one, suddenly being alone and maybe not being able to get out of the house. Maybe no one is visiting them, they become depressed and they lose their appetite’ (GHC 2).

They commonly encountered patients who refused to eat or drink for different reasons. Some patients did not want to eat because of problems going to the toilet or a fear of becoming constipated. Some had a different perception of their nutritional needs than the healthcare professionals and limited their food intake because they wanted to lose weight. Some patients would stop eating because they were tired of life and did not want to live any more. Some patients who were at the end of their lives did not see the point of nutritional treatment. Carers also found that some patients were reluctant to receive help with meals because they wanted to be in charge of their own lives: ‘Many say I am old now and I eat what I want’ (HC 4). They generally perceived that many of their patients were tired of being urged to eat by healthcare professionals and described having to decide between urging the patients to eat and respecting their refusal to eat.

Medication may negatively affect appetite, and the carers experienced that patients who took many medicaments were often reluctant to eat. In contrast, the participants highlighted how medication could positively affect a patient’s appetite when treating underlying symptoms like nausea and pain.

#### Seeing nutrition as a part of the whole

All the participants described nutritional care as an essential part of treatment and care and emphasised the need for a holistic approach in order to provide older patients with adequate nutritional care: ‘It is not just nutrition, I assess the whole situation, and I see that things are connected. I believe that is my strength’ (HC 3). They talked about seeing the whole person when dealing with nutritional problems and providing nutritional care based on the patients’ needs and preferences. They described how a poor nutritional status could negatively affect the patients’ health and increase the length of hospital stays. They also described slow wound healing as one of the most visible consequences of poor nutritional status.

Some participants described working toward a more positive focus of the nutritional care. One participant from home care said:

‘We work with the psychology behind it now. How can we turn food and drink into something positive. Think about the patients who are visited by us four times a day: “Have you eaten?” “Do you want me to make you some food?” “You have to eat!” They get so fed up by it – it becomes negative and just a hassle’ (HC 5).

They described how their first priority was to serve tasty meals rather than nutritious meals, because if the patients did not like the food they would not eat at all. They also found that the patients ate better when they socialised during meals. As one hospital nurse said, ‘I think we have become very good at getting the patients out of their rooms so they can sit together and eat. That’s much better, and they eat well. It is almost like they motivate each other to eat’ (GHC 9).

### The structure of the nutritional care

#### Nutritional routines- much ado, but for what?

Hospital healthcare professionals described an increased focus on nutritional care on their wards. Staff from both hospitals described that they had recently implemented new nutritional routines in line with the national recommendations. Both wards had a nurse who was especially responsible for implementing these routines:

‘She is responsible for the nutritional routines on the ward. So we have started making nutritional care plans, we calculate calories, nutritional drinks – we have developed our own meals, snacks for the patients who need extra calories, nutritional drinks, etc.’ (GHC 10).

Hospital staff described routines for nutritionally screening all the patients upon admission and assessing food intake and energy requirements throughout their stay. However, several carers described a lack of protocol following up on the results of these assessments:

‘We are very good at screening the patients and calculating their calorie intake so that we can compile a nutritional care plan, but we never make the plan . . . we calculate and calculate calories, but we don’t use the results of these calculations for anything’ (GHC 4).

Home care staff generally also experienced being highly focused on nutritional care, and many of them described procedures for performing nutritional care that were in line with the clinical guidelines. They reported having regular routines for nutritionally screening their patients: ‘We do regular nutritional assessments to follow the patients’ nutritional situations’ (HC 8). They also described the different nutritional measures they would use when working with malnourished patients: ‘We document food and fluid intake, have regular weightings, cook meals, keep them company during meals, provide them with nutritional drinks and we observe their general health’ (HC 2). However, similar to the hospital staff, home care staff experienced a lack of procedure for following up on screening results:

‘I think there are many shortcomings in the nutritional care. That’s how I feel, and I don’t know if I am right to say so, but I experience that we do nutritional screenings and find that the patient is undernourished, but what do we do? Not much. The most common thing we do is providing them with nutritional drinks and not much else’ (HC 1).

The hospital and home care staff reported that an extensive use of temporary personnel without proper training in their nutritional routines negatively affected the quality of nutritional care.

#### Lack of time to individualize nutritional care

A lack of time was a repeated issue among hospital and home care staff; they reported that this affected their ability to provide the patients with individualised nutritional care. Even though the hospital staff described having an extensive choice of meals to offer the patients, they did not always have time to ask the patients about their food preferences. They described their inability to offer the patients frequent meals because of time constraints. Lack of time also affected how much the hospital staff read and documented nutritional information in the patients’ charts. They described how this inadequate documentation resulted in a lack of essential nutritional information when communicating patient information to the next level of care:

‘We write a summary when they have home care, but we don’t write much about nutrition. We may write things like lack of appetite, doesn’t drink enough and needs to be motivated, but we don’t write anything about how to address these issues, like whether or not they need nutritional drinks, high-energy drinks or enriched food’ (GHC 4).

Hospital staff also experienced that nutritional care was considered to be of secondary importance compared to other more urgent healthcare aspects. At time of discharge, they described only having enough time to talk to the patient about the most important issues, such as medication and practical information about the transportation home.

Home care staff reported having large workloads and not enough time to fulfil their mandated responsibilities, resulting in inadequate nutritional care. They described how constantly being assigned new patients and the limited time allocated for each patient constrained their ability to interact with residents and build on the relational aspects of caregiving at mealtimes. As a result, they felt that the nutritional care became task- and routine-oriented. They also felt that the allocated time for general care and help with meals (they repeatedly gave 10–15 min as an example) for each patient was insufficient to tend to the patient’s nutritional needs. This resulted in having to negotiate overall care, which meant taking time away from some patients in order to help patients with more urgent needs.

#### Lack of interdisciplinary collaboration in nutritional care

When dealing with nutritional problems, hospital staff described working in an interdisciplinary way with dieticians, doctors, speech and language therapists and occupational therapists. However, some carers experienced being uncertain about each other’s roles in nutritional care and felt that the communication and collaboration between the different professionals should be better.

In contrast, the home care staff described being alone in nutritional care, as there was no one with nutritional competence they could consult with when they encountered challenging situations. As far as they knew, there was no dietician employed in their unit or in the municipality that they could contact. In general, the nutritional knowledge they had they acquired during their nursing education. Several of them talked about using common sense in nutritional care: ‘I use common sense. I’m a mother of two, and I feel that I have experience’ (HC 9*)*. If they wanted to increase their nutritional competence, they had to do this during their free time. If they were provided with courses in nutrition, these were often held by the companies producing nutritional products, such as nutritional drinks, who, of course, promoted their own products.

#### Meeting challenging situations alone with limited resources in home care

The home care staff explained how they had to deal with a variety of complex situations in the patients’ own homes that led to an adaptation of care to each unique situation. They described not having resources to delve into the complexity of the patient’s situation and adapt the care accordingly. Instead, they resorted to simple solutions such as nutritional drinks and ready meals. The food they prepared usually consisted of sandwiches and microwave meals, and they found that the patients expressed a dislike of this food:

‘The quality should have been much better. The dinners we serve consist of throwing a ready-meal in the microwave and then putting it on the table – that’s it! Many of our patients tell us that they don’t like these meals. They want homemade meals. We should definitely have had more time for cooking’ (HC 7).

The home care services granted to each patient are outlined in a decision letter, which specifies what type of care the patient has been granted and how much time is allocated for this care. The healthcare professionals described being bound to the content of this decision letter and felt that this restricted their ability to assess the patients’ situations and care needs and to adapt the care accordingly:

‘It is challenging. Many are very concerned with the written instructions for each patient. If they say, “clean and change diapers”, that’s the only thing they do, and then they leave, but we need to be more open and see the whole situation’ (HC 1).

Many of the participants from home care told stories of situations where they felt that the nutritional care had failed:

‘They hadn’t started any nutritional treatment or given her nutritional drinks. They said they had assessed the situation, and that the patient could manage on her own, but she weighed 39 kilos and had no food in the fridge, and she had just moved into a new flat in a new area’ (HC 1).

## Discussion

Our findings show that healthcare professionals meet with complex and challenging situations when providing nutritional care to older patients. Participants described the nutritional care in hospitals as highly prioritised, but inconsistent in terms of routines, lack of time and extensive use of temporary staff. In home care, the participants emphasised nutritional care as important; however, a lack of training, interdisciplinary support and time dedicated to food-related care limited the carers’ ability to provide quality nutritional care.

These findings are in line with previous studies in terms of the barriers to providing quality nutritional care to older patients [12, 14–16, 26]. These studies acknowledge healthcare professionals’ experiences of challenges, but they emphasise that the professionals can resolve many of these problems themselves by increasing their competence and awareness rather than contextualising their experiences in their larger institutional and organisational settings.

Healthcare professionals are not free to perform care as they wish. They work under organisational conditions and constraints that regulate their practice, and they are often torn between the demands of the care recipients to improve the responsiveness to their needs and the demands of their organisations to provide cost-effective and efficient care [18]. A systematic review by Fromholt Olsen et al. [27], found that home care providers experienced considerable ambivalence and stress related to providing care according to their older clients’ needs and preferences due to situations where they had to balance the needs of their older clients against their professional standards and the conflicting and competing demands of their organizations [27].

Both the hospital and home care staff described the need for a holistic approach to nutritional care in order to meet the older patients’ complex nutritional needs. They did, however find that they were unable to provide care according to these needs due to organisational conditions and limited resources. In particular, the home care staff found that this was challenging. They described having little time to get to know their patients and their needs. Additionally, their work was guided by clearly defined tasks and time schedules (i.e. the municipal decision letters), and they described how the limited resources to deal with the complexity of the situations resulted in care that was task- and routine-oriented.

Older patients are a diverse group, and each individual has unique nutritional needs and preferences. However, the routinisation of work makes it difficult for healthcare professionals to review needs and preferences and respond with individualised care [18, 28]. A previous study found that healthcare professionals found it demanding to address factors contributing to malnutrition, including social isolation, lack of supervision during meals and limited choice of food due to inadequate time allocated for nutritional care [16]. Moreover, Teeri et al. [29] found that nurses tended to disregard patients’ individual needs because the care they provided was regulated by strict routines and schedules. Another study reported that routine-driven practices constrained the quality of the psychosocial aspects of nutritional care [10].

Most of the healthcare providers described having set procedures for nutritional care. They performed regular weightings, nutritional screenings, documentation of food intake, etc.; however, this did not always seem to benefit the patients. For instance, they described spending time screening and calculating calorie intake, but they lacked procedures to follow up on these results. It seems that this is done because it is recommended as a best practice nutritional care in the clinical guidelines, without considering how this could be used to improve patient care. Clinical guidelines are the most common way to ensure the provision of equal and evidence-based treatment, and they can help to ensure safe nutritional care for patients. It is, however, important that healthcare providers are aware that clinical guidelines are guidelines, not rules, in order for them to give optimal nutritional care [30, 31]. However, guidelines have been criticised for failing to address the needs and preferences of older patients with complex health problems [32]. They also constitute a form of standardisation of professional clinical care, and the patients’ needs are probably not the only priority during the making of these recommendations, as they may also be influenced by the need to save costs, serve societal needs or to protect special interests [33–35]. Care should be based on the patient’s health needs and preferences and be adapted appropriately [30, 31, 35].

Many previous studies have suggested that developing healthcare professionals’ knowledge and skills in nutritional care will improve nutritional care practice [12, 14–16, 26]. Contrary to findings of poor knowledge about nutritional care among healthcare professionals in these previous studies, in the present study, the participants’ descriptions of how to perform nutritional care indicate that they possess rich knowledge about the topic. They did however; point out that temporary staff lacked the necessary nutritional knowledge. Suominen, Kivisto and Pitkala [36] found that nutritional education could improve practice and increase patients’ energy intake. Thus, increasing competence among healthcare providers seems to be a good initiative. However, knowledge alone is unlikely to result in practice changes as long as the municipal healthcare context in Norway is characterised by a mismatch between caring responsibilities and allocated resources [37]. Unless the organisational frames for providing care are addressed, increasing knowledge may just increase the gap between theory on the best practices in nutritional care and what is realistic in practice (and an increased feeling of professional inadequacy).

The home care staff also described lacking interdisciplinary support in nutritional care. Nutritional problems among older patients are often complex and are determined by physiological, psychological and social changes; they lead to poor appetite, insufficient food intake and poor health. The complexity of the problems makes treatment challenging and often requires a multifactorial and multidisciplinary approach [38]. Having available dieticians or staff with special nutritional competence to assist in challenging situations could increase the time for and quality of the nutritional care and be beneficial to the home care staff and their care recipients.

The hospital healthcare professionals described spending considerable resources on nutritional care. In 2016, the average length of a hospital stay in Norwegian hospitals was four days [39]. Treating malnutrition is, however, a prolonged process. In the home care setting, where many patients recover from a hospital stay and spend the majority of their time, there seem to be limited resources to follow up on the nutritional initiatives taken in hospital and to promote good nutritional health. In addition, studies report that poor communication and coordination of nutritional care between hospital and municipal services affects the quality of nutritional care [13, 40]. If the nutritional treatment from the hospital is not followed up at home, it is worth asking whether the time and effort spent on nutritional care during the short hospital stay is a sensible use of resources. It may be more demanding and costly to provide high-quality interdisciplinary care in the patients’ home than in an institution, where patients and resources are together under one roof. However, it is fair to assume that people who become malnourished and admitted to hospitals are even more costly [2, 41]. In addition, malnutrition and its consequences cause suffering and reduced quality of life for many older people [42]. It seems that the focus continues to be on the way healthcare professionals provide care’ and less on how organisations create structures and cultures that allow for person-centred care [17]. We need to begin a conversation about whether we as a society are comfortable with a situation in which older people are denied their human rights for proper nutritional care.

### Strengths and limitations

Although generalising qualitative findings is not a goal of qualitative research, qualitative researchers strive for the transferability of their findings. To address this, we aimed to provide rich and detailed descriptions of the healthcare providers’ experiences.

The hospital nurses were younger and had less experience in the practice field than the home care nurses. This may have influenced how they experienced providing nutritional care, and therefore, the findings in this study. The five healthcare providers recruited by their nursing managers may also have influenced the results. We cannot rule out the possibility that the managers recruited staff whom they considered particularly competent or whom they believed had a positive view of the services. The interviews took place in a secluded place at the participants’ work place where no one else could listen to the conversation. However, being in their own workplace may have restricted the healthcare professionals’ willingness to talk freely when it came to having critical views on the nutritional care practice. Further, the authors of this study are researchers with training in nursing (PhD), nutrition and health sciences (PhD candidate), clinical nutrition (PhD), public nutrition (PhD) and physiotherapy and health sciences (PhD) with extensive training and experience in conducting qualitative research; thus, our study design, data collection and data interpretation will have been influenced by our backgrounds and preconceptions. We discussed and reflected upon this throughout the research process.

## Conclusions

The healthcare professionals described having a high focus on and priority of nutritional care when caring for older patients. They experienced meeting challenging and complex nutritional problems among their older patients and expressed the need for an individualised and holistic approach to nutritional care. However, they did find it challenging to practice individualized nutritional care due to the complexity of the patients’ nutritional problems and constraints in the way nutritional care was organised. By describing the challenges the healthcare professionals face when trying to individualize the nutritional care, this study may provide important knowledge to health professionals and policy makers on how to decrease the gap between older patients’ preferences for care and nutritional care practice.

## Supplementary information

**Additional file 1.** Interview guide. The semi-structured interview guide used to direct the interviews with the healthcare professionals.

## Data Availability

The datasets analysed during the current study are not available. According to the ethical approval, only the members of the research team are granted access to these data sets.

## Abbreviations

COREQ: The consolidated criteria for reporting qualitative studies
GHC: Acute Geriatric hospital care
HC: Home care
REC: The Regional Committees for Medical and Health Research Ethics

## References

[1] Beck AM, Balkn UN, FÜRst P, Hasunen K, Jones L, Keller U (2001). Food and nutritional care in hospitals: how to prevent undernutrition–report and guidelines from the Council of Europe. Clin Nutr.

[2] Brotherton A, Simmonds N, Stroud M. Malnutrition matters. Meeting Quality Standards in Nutritional Care. BAPEN; 2010.

[3] Guttormsen AB, Hensrud A, Irtun Ø, Mowé M, Sørbye LW, Thoresen L, et al. Nasjonale faglige retningslinjer for forebygging og behandling av underernæring. [National professional guidelines on prevention and treatment of malnutrition. Oslo 15–1580]. Oslo: The Norwegian Directorate of Health; 2013. Report No.: IS-1580.

[4] Kondrup J, Allison SP, Elia M, Vellas B, Plauth M (2003). Educational, et al. ESPEN guidelines for nutrition screening 2002. Clin Nutr.

[5] Volkert D, Beck AM, Cederholm T, Cruz-Jentoft A, Goisser S, Hooper L (2019). ESPEN guideline on clinical nutrition and hydration in geriatrics. Clin Nutr.

[6] Volkert D, Berner YN, Berry E, Cederholm T, Coti Bertrand P, Milne A (2006). ESPEN guidelines on enteral nutrition: geriatrics. Clin Nutr.

[7] Volkert D, Chourdakis M, Faxen-Irving G, Fruhwald T, Landi F, Suominen MH (2015). ESPEN guidelines on nutrition in dementia. Clin Nutr.

[8] National Institute for Health and Clinical Excellence (2006). Nutrition support for adults: oral nutrition support, enteral tube feeding and parenteral nutrition.

[9] Bonetti L, Terzoni S, Lusignani M, Negri M, Froldi M, Destrebecq A (2017). Prevalence of malnutrition among older people in medical and surgical wards in hospital and quality of nutritional care: a multicenter, cross-sectional study. J Clin Nurs.

[10] Dunn H, Moore T (2016). 'You can't be forcing food down 'em': nursing home carers' perceptions of residents' dining needs. J Health Psychol.

[11] Eglseer D, Halfens RJ, Lohrmann C (2017). Is the presence of a validated malnutrition screening tool associated with better nutritional care in hospitalized patients?. Nutrition..

[12] Eide HD, Halvorsen K, Almendingen K (2015). Barriers to nutritional care for the undernourished hospitalised elderly: perspectives of nurses. J Clin Nurs.

[13] Halvorsen K, Eide HK, Sortland K, Almendingen K (2016). Documentation and communication of nutritional care for elderly hospitalized patients: perspectives of nurses and undergraduate nurses in hospitals and nursing homes. BMC Nurs.

[14] O’Connell MB, Jensen PS, Andersen SL, Fernbrant C, Nørholm V, Petersen HV (2018). Stuck in tradition-a qualitative study on barriers for implementation of evidence-based nutritional care perceived by nursing staff. J Clin Nurs.

[15] Beattie E, O’Reilly M, Strange E, Franklin S, Isenring E (2014). How much do residential aged care staff members know about the nutritional needs of residents?. Int J Older People Nursing.

[16] Watkinson-Powell A, Barnes S, Lovatt M, Wasielewska A, Drummond B (2014). Food provision for older people receiving home care from the perspectives of home-care workers. Health & social care in the community.

[17] McCormack B, McCance T, Klopper H. Person-centred practice in nursing and health care : theory and practice. West Sussex, England: Wiley Blackwell; 2017.

[18] Lipsky M. Street-Level Bureaucracy. Dilemmas of the Individual in Public Services: Russell Sage Foundation; 2010.

[19] Thorne S. Interpretive description. USA: Left Coast Press; 2008. 272 p.

[20] Braun V, Clarke V (2006). Using thematic analysis in psychology. Qual Res Psychol.

[21] Creswell JW, Poth CN. Qualitative inquiry & research design : choosing among five approaches. Fourth edition. ed. Los Angeles: SAGE; 2018. xxv, 459 pages p.

[22] Wollscheid S, Eriksen J, Hallvik J (2013). Undermining the rules in home care services for the elderly in Norway: flexibility and cooperation. Scand J Caring Sci.

[23] Eide T, Nilsen ER, Gullslett MK, Olafsen AH, Aaberge AH, Eid H. Tillitsmodellen – erfaringer med mini-pilotering av selvstyrende team i tre bydeler i Oslo kommune. Drammen, Norway: Høgskolen i Sørøst-Norge Vitensenteret Helse og teknologi 2017. Contract No.: 13 / 2017.

[24] Lincoln YS, Guba EG (1985). Naturalistic inquiry.

[25] Tong A, Sainsbury P, Craig J (2007). Consolidated criteria for reporting qualitative research (COREQ): a 32-item checklist for interviews and focus groups. Int J Qual Health Care.

[26] Bonetti L, Bagnasco A, Aleo G, Sasso L (2013). The transit of the food trolley’- malnutrition in older people and nurses’ perception of the problem. Scand J Caring Sci.

[27] Olsen CF, Bergland A, Debesay J, Bye A, Langaas AG. Striking a balance: health care providers’ experiences with home-based, patient-centered care for older people—a meta-synthesis of qualitative studies. Patient Educ Couns. 2019. 10.1016/j.pec.2019.05.017 31160128

[28] Freidson E. Professionalism. The third logic. UK: Polity Press; 2001.

[29] Teeri S, Leino-Kilpi H, Välimäki M (2006). Long-term nursing Care of Elderly People: identifying ethically problematic experiences among patients, relatives and nurses in Finland. Nurs Ethics.

[30] Timmermans S (2005). From autonomy to accountability: the role of clinical practice guidelines in professional power. Perspect Biol Med.

[31] Timmermans S, Kolker ES (2004). Evidence-based medicine and the reconfiguration of medical knowledge. J Health Soc Behav.

[32] Boyd CM, Darer J, Boult C, Fried LP, Boult L, Wu AW (2005). Clinical practice guidelines and quality of Care for Older Patients with Multiple Comorbid DiseasesImplications for pay for performance. JAMA..

[33] Boudoulas KD, Leier CV, Geleris P, Boudoulas H (2015). The shortcomings of clinical practice guidelines. Cardiology..

[34] Gnant Michael (2013). Guidelines: Usefulness and Limitations. Breast Care.

[35] Sheridan DJ, Julian DG (2016). Achievements and Limitations of Evidence-Based Medicine. J Am Coll Cardiol.

[36] Suominen MH, Kivisto SM, Pitkala KH (2007). The effects of nutrition education on professionals practice and on the nutrition of aged residents in dementia wards. Eur J Clin Nutr.

[37] Halvard Vike JD, Haukelien H (2016). Tilbakeblikk på velferdsstaten. Politikk, styring og tjenester.

[38] Landi F, Calvani R, Tosato M, Martone AM, Ortolani E, Savera G (2016). Anorexia of aging: risk factors, consequences, and potential treatments. Nutrients..

[39] Statistics Norway. Døgnopphold, liggedager og gjennomsnittlig liggetid ved somatiske sykehus, etter kjønn (Overnight stays, and average length of stay in somatic hospitals, by gender): Statistics Norway; [Available from: https://www.ssb.no/304492/dognopphold-liggedager-og-gjennomsnittlig-liggetid-ved-somatiske-sykehus-etter-kjonn-sa-134.

[40] Holst M, Rasmussen HH (2013). Nutrition therapy in the transition between hospital and home: an investigation of barriers. J Nutr Metab.

[41] Elia M. The cost of malnutrition in England and potential cost savings from nutritional interventions (full report). Malnutrition Action Group of BAPEN and National Institute for Health Research Southampton Biomedical Research Centre 2015.

[42] Leslie Wilma, Hankey Catherine (2015). Aging, Nutritional Status and Health. Healthcare.

