# Supplementary material for: Healthcare professionals’ experiences of providing individualized nutritional care for Older People in hospital and home care: a qualitative study
Source: BMC Geriatr. 2019 Nov 20;19:317. doi: 10.1186/s12877-019-1339-0 (PMC6865038; doi:10.1186/s12877-019-1339-0)
Supplement: Supplementary file 1 — Additional file 1. Interview guide. The semi-structured interview guide used to direct the interviews with the healthcare professionals. [file 12877_2019_1339_MOESM1_ESM.docx]

**Additional file 1: Interview guide**

Can you please tell me how you perceive older patients’ needs for nutritional care?

Can you describe how you practice nutritional care in your ward/unit?

How do you perceive the quality of the nutritional care you provide?

How do you perceive your knowledge about nutritional care to older persons?

There are best practice guidelines for how to practice nutritional care, how do you apply these in your unit/ward?

Can you describe your experiences with nutritional screening?

If applicable: How do you apply the screening results in the nutritional treatment?

Can you describe what measures you apply when patients are identified to be at nutritional risk or malnourished?

Can you describe how you cooperate with other professions about the nutritional care?

How do you experience that the patients’ needs and preferences are met in the nutritional care you provide?

Can you describe factors that facilitate or prevent you from providing patients with nutritional care according to their needs?

Do you have any thoughts about how the nutritional care for older patients may be improved in hospital/home care?
